# Evolutionary responses to codon usage of horizontally transferred genes in *Pseudomonas aeruginosa*: gene retention, amelioration and compensatory evolution

**DOI:** 10.1099/mgen.0.000587

**Published:** 2021-06-24

**Authors:** Martijn Callens, Celine Scornavacca, Stéphanie Bedhomme

**Affiliations:** ^1^​ CEFE, Univ Montpellier, CNRS, EPHE, IRD, Univ Paul Valéry Montpellier 3, Montpellier, France; ^2^​ Institut des Sciences de l’Evolution, Université Montpellier, CNRS, IRD, EPHE, Montpellier, France

**Keywords:** codon usage, compensatory evolution, gene amelioration, gene retention, horizontal gene transfer, *Pseudomonas aeruginosa*

## Abstract

Prokaryote genome evolution is characterized by the frequent gain of genes through horizontal gene transfer (HGT). For a gene, being horizontally transferred can represent a strong change in its genomic and physiological context. If the codon usage of a transferred gene deviates from that of the receiving organism, the fitness benefits it provides can be reduced due to a mismatch with the expression machinery. Consequently, transferred genes with a deviating codon usage can be selected against or elicit evolutionary responses that enhance their integration, such as gene amelioration and compensatory evolution. Within bacterial species, the extent and relative importance of these different mechanisms has never been considered altogether. In this study, a phylogeny-based method was used to investigate the occurrence of these different evolutionary responses in *Pseudomonas aeruginosa*. Selection on codon usage of genes acquired through HGT was observed over evolutionary time, with the overall codon usage converging towards that of the core genome. Gene amelioration, through the accumulation of synonymous mutations after HGT, did not seem to systematically affect transferred genes. This pattern therefore seemed to be mainly driven by selective retention of transferred genes with an initial codon usage similar to that of the core genes. Additionally, variation in the copy number of tRNA genes was often associated with the acquisition of genes for which the observed variation could enhance their expression. This provides evidence that compensatory evolution might be an important mechanism for the integration of horizontally transferred genes.

## Data Summary

Our analysis is based on full genome assemblies of *Pseudomonas aeruginosa* strains that are publicly available on NCBI GenBank. Table S1 (available in the online version of this article) contains all accession numbers of the genome assemblies used in this study. The scripts used to perform the analyses described in this study can be found here: https://github.com/MartijnCallens/P.aeruginosa_HGTCodonUse

**Impact Statement**

This study considers the importance of codon usage for horizontal gene transfer on a long-term evolutionary scale. In this context, different evolutionary processes have been hypothesized to occur (selective gene retention, gene amelioration and compensatory evolution). Evidence for these processes is, however, scarce, and they have never been simultaneously considered. This study addresses this gap in the literature and shows that multiple processes might interact for the long-term integration and evolution of horizontally transferred genes. Our findings have strong implications for the acquisition of important traits through horizontal gene transfer, such as antibiotic resistance and increased virulence, in the opportunistic pathogen *Pseudomonas aeruginosa*. The observed patterns are furthermore not expected to be limited to *P. aeruginosa,* but can be widespread in bacteria.

## Introduction

Horizontal gene transfer (HGT) strongly contributes to bacterial evolution and is a key mechanism for a quick adaptation to environmental challenges. The frequent uptake of foreign genetic material leads to numerous genes being found in certain genomes of a bacterial species, but not in all. This set of dispersed genes, the accessory genome, is highly dynamic and is shaped by frequent gene gains and losses [1]⁠. A new gene is incorporated into the accessory genome if – after initial acquisition – it replicates itself within the receiving organism and is vertically inherited. However, long-term retention of a gene will depend on its effect on the fitness of the receiving organism, which is often dependent on the environmental and genomic context. For a gene to be retained over evolutionary time, the cost of replicating the transferred genetic material and expressing any genes contained in it has to be counterbalanced by a net fitness benefit. Otherwise, there is a high probability that it will be subsequently lost due to natural selection [2]⁠.

One factor that can influence the cost of expressing a horizontally acquired gene is the degree of match of its codon usage to the translation machinery of the organism expressing it [3]⁠. Within a species, synonymous codons are often not used at equal frequencies but particular codons are preferred over synonymous alternatives to encode a same amino acid [4]⁠. These codon usage preferences are a well-documented phenomenon in bacteria and can be strongly differentiated between species [5]⁠. Although neutral processes can shape codon usage bias due to a species-specific mutational bias, in unicellular organisms with large effective population sizes there is often a selective component involved [6]⁠. In bacteria there is usually a good correlation between tRNA gene copy number and the frequency of corresponding codons, and preferred codons are generally more efficiently and accurately translated than less frequently used ones because of a higher degree of match to the available tRNA pool [7, 8]⁠. Consequentially, genes using rare codons often produce a low amount of functional protein due to slower translation, translation errors, protein truncation or protein misfolding [9]⁠. Furthermore, mRNAs containing non-optimal codons are found to be less stable than their synonymous equivalents using optimal codons [10]⁠. Sequestration of ribosomes on the mRNA is also higher when elongation rates are slow, reducing ribosome availability and global protein synthesis [2]⁠.

Horizontally acquired genes with a codon usage that is divergent from the receiving organism will thus potentially have lower relative fitness effects than when codon usage is similar. Medrano-Soto *et al*. showed that for multiple bacterial species, recently transferred genes predominantly exhibit a codon usage that is typical for the recipient genome [11]⁠. Such a gene retention bias can furthermore be correlated with the capacity of receiving organisms to translate these genes [12]⁠. It is not known if selection for gene retention only affects the probability of the initial uptake of transferred genes, or if this also affects the probability of gene retention over the long term. Moreover, if a gene with a divergent codon usage is retained after HGT, it is expected to evolve towards a more typical codon usage in a process called *amelioration* [13]⁠. This process relies on synonymous mutations accumulating in the transferred gene, and is assumed to be very slow when it is only driven by the local mutational bias. When selection on codon usage is involved, this process can occur faster and will depend on the fitness effects of individual synonymous mutations. Both selection against genes with a deviating codon usage and gene amelioration will cause accessory genes to exhibit a codon usage similar to that of the core genes over time. However, the temporal scale and relative contribution of both mechanisms has never been investigated within a bacterial species.

Another evolutionary mechanism to accommodate for a transferred gene with divergent codon usage is *compensatory evolution* (i.e. mutations elsewhere in the genome that reduce the cost associated to the transferred gene [14]). This mechanism has been shown to play an important role in preventing plasmid loss and the fixation of accessory traits in the chromosome [15]⁠. A potential strategy for compensatory evolution to alleviate the cost of divergent codon usage in transferred genes is increasing the availability of tRNAs that recognize the rare codons they contain [16]⁠. One way to do this is by increasing the gene copy number of these tRNAs, which is known to be correlated with cellular tRNA concentrations [17]⁠. McDonald *et al*. found evidence that this strategy is used to compensate for the influx of rare codons in the *E. coli/Shigella* complex [18]⁠. They showed that increases in tRNA gene copy number in *Shigella* and pathogenic *E.coli* O157:H7 strains was related to an overall increase of corresponding codons through the proliferation of selfish genetic elements or HGT. However, estimates on the frequency and extent of this mechanism are limited, especially because the co-occurrence of changes in tRNA copy number and codon frequencies through HGT has never been investigated in a phylogenetic explicit manner on a species-wide scale.

*Pseudomonas aeruginosa* is an ecologically versatile opportunistic pathogen that frequently acquires genes through HGT. This is reflected by the large number of genes found in its accessory genome, which are often acquired as genomic islands (i.e. functionally related clusters of genes inserted at specific genomic locations). Important phenotypic traits displayed by some strains, such as increased pathogenicity, are known to be related to the acquisition of genomic islands through HGT [19]⁠. Given the high %GC of *P. aeruginosa*, genes being acquired from other species would generally have a lower %GC, but this difference may decrease over time after integration in the genome [20]⁠. In this study, we simultaneously investigated the occurrence of three evolutionary responses related to the codon usage of transferred genes: selective gene retention, gene amelioration and compensatory evolution. To this end, we applied a phylogeny-based method to detect HGT events during the evolutionary history of *P. aeruginosa*. We evaluated the correlation between the time since a HGT event and the codon usage of the transferred genes. We then used ancestral sequence reconstruction to investigate if changes in codon usage were caused by mutational processes. We further investigated if variation in tRNA gene content corresponded to the influx of corresponding codons through HGT.

## Methods

A schematic overview of the bioinformatic pipeline used in this study is provided in Fig. S1.

### Genome sequences

An assembly search was done on ncbi.nlm.nih.gov/assembly (8 September 2017) for ‘*Pseudomonas aeruginosa*’. Status=‘latest RefSeq’ and Assembly level=‘complete genome’ were selected. This resulted in 96 available full genome assemblies. Two strains were subsequently removed from the dataset. Strain PA7 (GenBank accession GCF_000017205) was removed because it was found to be a taxonomic outlier (see also [21]). Possibly, mutation rates in this strain differ strongly from other strains, making branch length comparison and divergence time with other strains unreliable. Strain H47921 (GenBank accession GCF_001516345) was removed because it was found to be highly recombinant, making a correct phylogenetic placement of this strain difficult. Table S1 provides a list of the genomic sequences included in this study.

Genomes were re-annotated using PROKKA 1.12 [22] to obtain a consistent annotation across all genomes. All subsequent analyses were performed on genes predicted by PROKKA. tRNA genes were predicted using tRNAscan-SE 2.0.5 [23].

### Pan-genome analysis

An all-against-all nucleotide blast was performed on the full set of genes using blastn 2.2.31+. To cluster genes into groups of homologues, blast results were used as input for SiLiX 1.2.9 [24]⁠ with the following parameter values: overlap=0.7 (min. % overlap to accept blast hits for building families=70 %) and identity=0.6 (min. % identity to accept blast hits for building families=60 %). In parallel, the genome dataset was analysed with the Roary pipeline [25] to obtain an additional pan-genome analysis and to use its orthology assignments for splitting up large gene clusters produced by SiLiX.

Our goal here was not to define the *minimal* core genome of *P. aeruginosa,* but to obtain genes for constructing a strain phylogeny and to determine patterns of codon usage for genes expected to be in equilibrium with forces shaping codon usage. Therefore, we adopted a looser definition of core genes to maximize the number of genes included in this dataset, which is crucial to reconstruct a meaningful strain phylogeny in light of the similarity of the strains. This is expected to result in a larger number of core genes compared to other estimates [26]⁠. Single copy homologous gene clusters predicted by SiLiX present in at least 95 % of all strains (i.e. between 89 and 94 strains) were directly assigned as core genes. Multi-copy clusters predicted by SiLiX present in at least 95 % of all strains were split up into single-copy clusters using the orthology assignments produced by Roary, and were additionally assigned as core genes.

The remaining homologous gene clusters were assigned as accessory genes. Different reasons could have caused these genes to be classified as accessory genes: HGT, multiple deletions of ancestral core genes and erroneous classification of genes in a separate cluster due to, for example, gene duplication followed by sequence evolution leading to pseudogenization or sequencing and assembly errors (here termed *pseudo-accessory genes*). As only accessory genes that originated through HGT were relevant for our analysis, we filtered out accessory genes that originated through multiple gene deletions and pseudo-accessory genes. To filter pseudo-accessory genes, all accessory gene clusters were blasted against the full set of genes. Only those gene clusters that did not return any significant hits against members of other clusters were retained for further analysis. Details on filtering accessory genes that potentially originated through multiple deletions of ancestral core genes are described under the section ‘Inference of horizontal gene transfers’.

### Strain phylogeny

All core genes were aligned using mafft 7.271 with the ‘auto’ option [27]⁠. A core gene nucleotide alignment supermatrix was produced by concatenating all aligned core genes. The core genes' nucleotide alignment was used instead of the protein alignment because core-gene protein sequences were highly conserved among strains, making it difficult to obtain a good phylogenetic signal from the protein sequences. A maximum-likelihood phylogenetic tree was constructed with IQ-Tree 1.6.5 [28]⁠ using a GTR+F+R9 model of substitution chosen according to Bayesian information criterion obtained with ModelFinder [29]⁠. Branch support was obtained with 1000 bootstrap replicates. The strain phylogeny was rooted at midpoint. The position of the root was further supported by several potential placements of *P. pseudoalcaligenes* as the outgroup close to the midpoint with the evolutionary placement algorithm [30]⁠ and minimal ancestor deviation rooting, also placing the root at the midpoint [31]⁠.

### Inference of horizontal gene transfers

The overall small number of genes and low amount of sequence variation within homologous clusters of accessory genes did not allow us to construct reliable gene trees, preventing analysis of HGT based on phylogeny comparison (reviewed in [32]). Therefore, we used ancestral state reconstruction to infer HGT events. For each accessory gene, we constructed a phyletic pattern with gene copy number as character states. The probability of each character state was inferred for all nodes on the strain phylogeny using ancestral state reconstruction with stochastic character mapping. This was done using the Phytools R package running 1000 simulations for each character under the equal rate model. We used the probabilities of presence at the nodes of the strain phylogeny to infer HGT events. Horizontal transfer of an accessory gene was predicted to have occurred along a branch on the strain phylogeny if the probability of presence of this gene was lower than 0.5 on its parent node (most probable gene copy number=0) and higher than 0.5 on its child node (most probable gene copy number >0). To verify results obtained by Phytools, we additionally inferred HGT events with ancestral reconstruction by posterior probabilities in a phylogenetic birth-and-death model using the count program [33]⁠. This analysis inferred the majority of transfers on exactly the same branch (76.7 %). Despite some differences between Phytools and count, general patterns in subsequent analyses were highly similar using inferred HGTs from either program, indicating the robustness of our results. We therefore only included results obtained from HGT’s inferred by Phytools in this study.

Multiple independent gene losses after a single HGT event could produce a scattered phyletic pattern for which multiple false-positive HGT events are inferred. To correct for this, we used information on the genomic position of genes for which multiple transfers were inferred. Genes located at the same genomic position (inserted between the same core genes) were assumed to be acquired at the same moment, and their HGT was traced back to the last common ancestor of all separate HGT events inferred for this gene. We removed genes from the analysis that were inferred to be present at the root because their classification as accessory genes seemed to be caused by multiple losses of a gene that was present in the ancestor of all our strains.

Each gene that was acquired through HGT was assigned a ‘residence time’. This residence time was obtained from the strain phylogeny by taking the branch length from the leaf node of the strain containing this gene to the midpoint of the branch where the HGT was inferred. Residence time thus indicates the amount of evolutionary change that occurred in the core genome since a HGT event. We expect this metric to be correlated with actual time, but due to the lack of bacterial fossils, we were unable to fit a molecular clock. Furthermore, it also takes into account the variation in evolutionary rates in the core genome along a branch due to a higher mutation rate or strong selection, something that is also expected to affect the accessory genes contained in the genome.

### Codon usage

The codon usage of the core genome was determined by constructing a codon usage table based on all core genes using the cusp tool (EMBOSS). Metrics regarding codon usage of both core genes and HGT genes were calculated using the Codon Usage Similarity Index (COUSIN) tool [34]⁠ with the codon usage table of the core genome used as a reference. Codon usage metrics were only calculated for genes having at least 100 codons to avoid biases caused by gene length. The COUSIN [34] was used as a metric to determine how similar the codon usage of a gene was compared to the average codon usage in the core genome. This metric incorporates both the direction of codon usage bias (positive values represent a bias in the same direction as the reference, negative values are in the opposite direction), analogous to the Codon Adaptation Index (CAI [35]), and the strength of codon usage bias (indicated by the absolute values, with 0 indicating the absence of a bias and values above 1 indicating a stronger bias than the reference), analogous to the effective number of codons (ENC [36]), thus providing more information than each separate metric.

Overall change in COUSIN values for HGT genes in function of residence time was evaluated using a non-parametric Kendall’s rank correlation. Changes in minimal, maximal and median COUSIN values were evaluated by obtaining these values for each residence time interval of 0.001 and fitting a linear model. We additionally analysed changes in CAI values in function of residence time for comparison. There was a degree of uncertainty if genes inferred to be acquired near the root represented actual HGT events or were ancestral genes with an early loss in one clade (although less probable based on ancestral state reconstruction). Therefore, we also evaluated changes in COUSIN values in function of residence time when excluding HGT genes acquired along branches departing from the root (excluding 159 genes; 6.7 % of potential HGTs).

To evaluate the potential importance of gene amelioration for changes in codon usage in function of residence time, we inferred ancestral sequences of HGT genes using prank v.170427 by aligning extant sequences using the codon model and the core genome phylogeny as a guide tree. To allow for a sufficient amount of sequence variation when inferring ancestral sequences, this analysis was performed on a subset of 22 gene clusters that contained 20 or more extant sequences derived from the same HGT event. The sequence inferred at the ancestral node of the guide tree was used to determine the ancestral codon usage bias. Any gaps in the alignments were removed before assessing their codon usage bias. Significance of change in codon usage bias between the extant genes and their ancestral state was assessed using a one-sample *t*-test. Genes for whom the mean change in codon usage bias significantly differed from zero were considered to show directional evolution. We additionally assessed the significance of changes in codon usage bias caused only by synonymous variation by converting all non-synonymous mutations back to their ancestral state. This same analysis was performed on 78 gene clusters that contained 10 or more extant sequences, to test for the dependence of the results on our choice of threshold for minimal number of extant sequences in a gene cluster.

### Evolution of tRNA gene copy numbers

The tRNA gene content was inferred for the parent and child node of each branch on the strain phylogeny using ancestral state reconstruction based on the extant tRNA gene copy numbers found in each strain (same model as for accessory genes). We further estimated the genomic content of protein coding sequences for the parent and child nodes of each branch based on the predicted core genome content and ancestral state reconstruction of HGT genes.

To analyse compensatory evolution on a codon level, codon usage tables were obtained for the parent and child node of each branch based on the inferred genomic content. Changes in codon frequencies due to HGT (expressed as changes in the expected number of a codon per 1000 bases) were calculated by subtracting parent node codon frequencies from child node codon frequencies. A permutation test was used to evaluate if increments in tRNA gene copy number were associated with strong increases in the frequency of codons these tRNAs can translate [18]⁠. This was done by ordering changes in codon frequencies of all branches in descending order and assigning a rank number based on their position. The sum of rank numbers of changes in codon frequencies associated with an increase in the tRNA gene having a Watson–Crick base pairing was taken (=observed value). Rank numbers were subsequently randomized using 10^4^ permutations and a *P*-value was calculated based on the fraction of randomizations for which a rank value sum equal or lower than the observed value was obtained. The same analysis was performed separately for codons associated with increases in tRNA genes having wobble- and less-favoured wobble base pairing [37]⁠.

To analyse compensatory evolution on a gene level, we first calculated the absolute adaptiveness [38] of each codon for the parent and child nodes of branches along which a change in tRNA gene content was observed. The absolute adaptiveness of a codon *i*, denoted as *W_i_* was defined as

,$$W_{i}=\sum_{j=1}^{n_{i}}\left(1-s_{ij}\right){tGCN}_{ij}$$

with *n_i_* being the number of tRNA isoacceptors that recognize the *i*th codon, tGCN*_ij_* the gene copy number of the *j*th tRNA that recognizes the *i*th codon, and *s_ij_* a selective constraint on the efficiency of the codon-anticodon pairing. Optimized *s*_*ij*_ values for *P. aeruginosa* were obtained from [39]⁠. The expected translation efficiency of a gene in a given tRNA context is then calculated as the geometric mean of the absolute adaptiveness of its codons. This metric is highly similar to the tRNA Adaptation Index (tAI) [38]⁠, which was designed to compare different genes to the same tRNA pool and uses the relative adaptiveness of codons instead of the absolute adaptiveness. However, tAI was not directly applicable to our question of comparing the translation efficiency of the same genes in the context of a variable tRNA pool because duplications in already favoured tRNA isoacceptors caused either no change or a lowering of the tAI value of all genes, which is biologically not meaningful. Using the absolute adaptiveness instead of relative adaptiveness of codons solved this issue. Because of its high similarity to tAI, we will refer to this metric as tAI_abs_. Change in the tAI_abs_ of each gene was calculated by subtracting the tAI_abs_ obtained with the tRNA gene content at the parent node from the tAI_abs_ obtained with the tRNA gene content at the child node (ΔtAI_abs_). For each branch where the tRNA gene content variation caused a positive mean ΔtAI_abs_, a non-parametric Mann–Whitney U-test was used to evaluate if the ΔtAI_abs_ differed significantly between HGT genes acquired along that branch and genes already present at the parent node (core genes and HGT genes acquired at an earlier time).

## Results

### Pan-genome analysis, strain phylogeny and core genome codon usage

A pan-genome analysis was performed to cluster homologous genes, and classify them as either core or accessory genes. This analysis gave a total of 16 007 distinct clusters of homologous genes. From them, 5288 were classified as core genes, based on the numbers of strains they occurred in (4330 genes occurred in all 94 strains and 958 genes occurred in between 89 to 93 strains). A total of 10 719 homologous gene clusters were present in less than 89 strains and were classified as accessory genes.

**Fig. 1.**
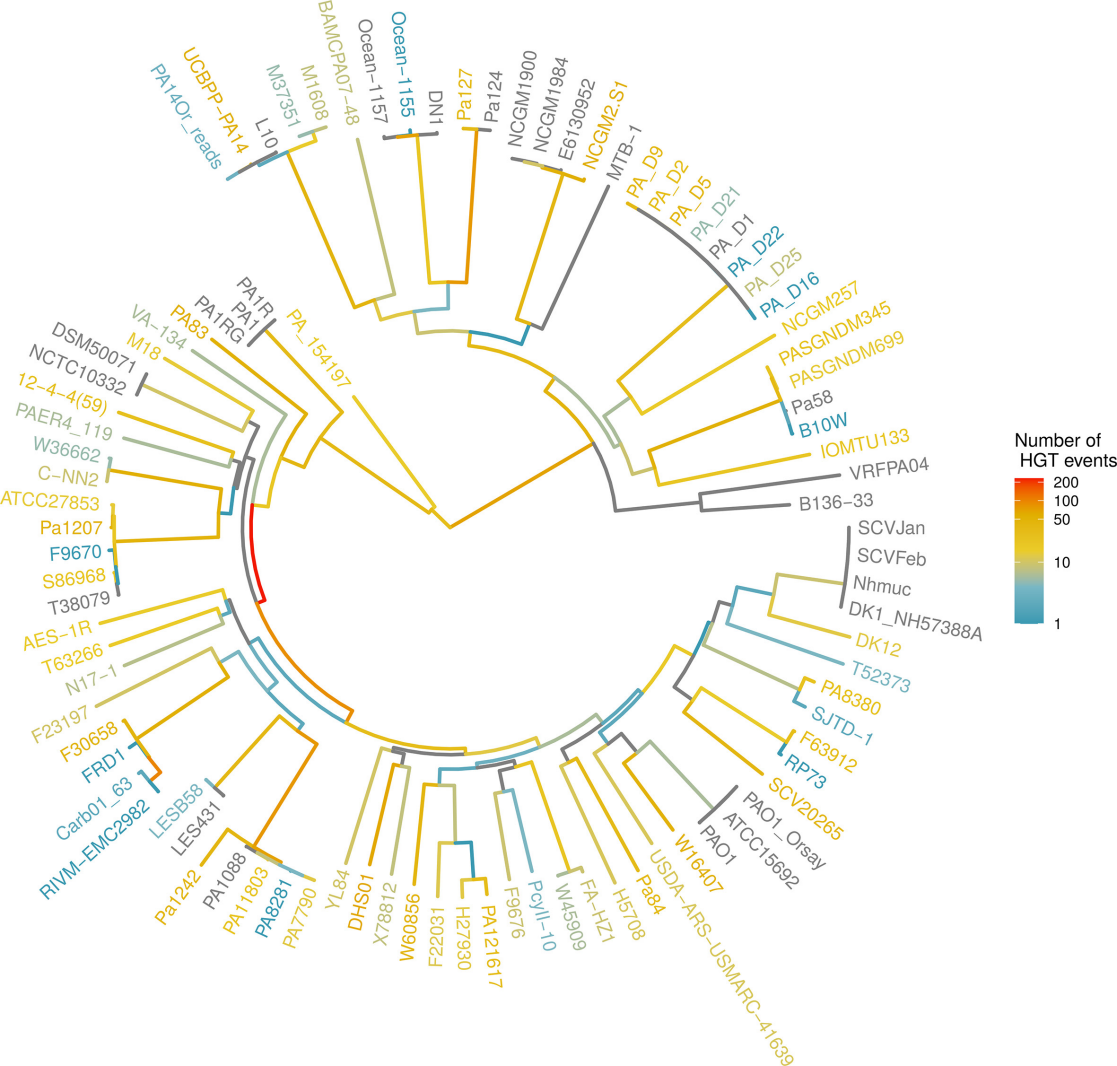
Number of HGT events along the evolutionary history of *P. aeruginosa*. The phylogeny of *P. aeruginosa* was determined using the alignment of the core genes. Branch colour indicates the number of HGT events detected along each branch. The colour of the tip labels give an indication of the number of HGT genes that were unique to a strain.

We constructed a phylogeny of the *P. aeruginosa* strains used in this study based on the nucleotide sequence alignment of their core genes. This phylogeny indicated a structure consisting of two large clades, with 61 strains belonging to the clade containing reference strain PAO1 and 32 strains belonging to the clade containing reference strain PA14 (Fig. 1). Strain PA154197 branched off close to the root and did not seem to be closely related to either one of these two clades. Although bootstrapping indicated a good support for this general structure, some smaller clades were found to be less-well supported (Fig. S2).

**Fig. 2.**
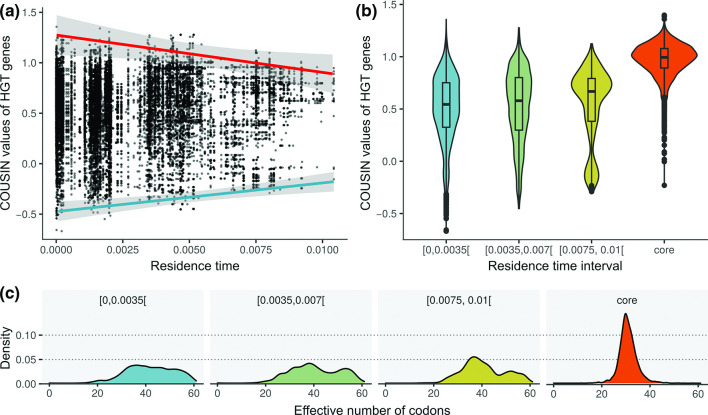
Codon usage bias of HGT genes in relation to their residence time. (a) Scatterplot of the COUSIN value of HGT genes in function of their residence time. The COUSIN value incorporates both the direction of codon usage bias (positive values represent a bias in the same direction as the reference, negative values are in the opposite direction) and the strength of codon usage bias (indicated by the absolute values, with 0 indicating the absence of a bias and values above 1 indicating a stronger bias than the reference). The blue and red regression lines were determined by taking respectively the minimal and maximal COUSIN value for each residence time interval of 0.001. (b) Violin plot showing the distribution of COUSIN values of HGT genes for each residence time interval of 0.0035 and the core genes. (c) Density plots showing the distribution of the ENC for HGT genes for each residence time interval of 0.035 and for the core genes. ENC values range between ranges between 20 (only one codon is used for encoding each amino acid) and 61 (all synonymous codons are used more or less equally).

Analysis of the GC-content of the core genome gave an average of 67.4 % GC for coding regions and 62.0 % GC for intergenic regions. The GC-content of full genomes was slightly lower than the core genome (66.4 % GC for coding regions and 60.7 % GC for intergenic regions). The construction of a codon usage table for the core genes showed that the codon usage in core genes reflected this relatively high % GC, where G or C nucleotides were always favoured over A or T on degenerate first and third codon positions (Table S2). Core genes generally had a strong codon usage bias, as shown by their overall low ENC values (mean=30.8±3.8; Fig. 2c).

### A large fraction of accessory genes was inferred to be acquired through HGT

We compared each accessory gene cluster to all other non-homologous gene clusters to filter out pseudo-accessory genes (i.e. accessory genes that most likely originated from genes that were already present in the genome). With this analysis we identified a large fraction of accessory genes that showed evidence of pseudogenization (33.4 % of all accessory genes) based on their low sequence overlap (<70 %) but high shared sequence identity (>90 %) with a different non-homologous gene cluster. One-third of these putative pseudo-accessory genes were partial sequences of larger genes that occurred in more than 89 strains, and could thus be considered truncated versions of duplicated core genes (11.5 % of all accessory genes). The remaining putative pseudo-accessory genes were mainly length variants of genes occurring in only a limited number of strains. Errors in the sequencing and assembly steps could also be the cause of these short overlaps. However, a few cases involved genes with high similarity to non-homologous genes because of shared conserved domains (e.g. conserved domains in the phosphate-selective porin O and P family). Several genes present on known genomic islands were also filtered out during this step, despite strong indications for a HGT origin (e.g. only 19 out of 32 genes on the LESGI-1 genomic island were classified as HGT). Although these latter cases could represent genuine non-homologous clusters and HGTs, they were not retained for further analysis because differences between these cases and pseudo-accessory genes were often not clear-cut and we opted for a stringent filtering to avoid false positives.

After applying the previous filtering step, we performed an ancestral state reconstruction for all the remaining accessory genes. This analysis inferred that 6.9 % of the accessory genes were present in the common ancestor of all strains included in this dataset, indicating that these genes were classified as accessory genes because of multiple deletions throughout the evolutionary history of *P. aeruginosa*. Further evidence for multiple deletions of genes present in the common ancestor was provided by the large number of genes that were found at the same genomic location, but with a highly dispersed occurrence on the strain phylogeny (545 genes were present in only a small number of strains on both sides of the root at the same genomic location).

As in our analysis we were only concerned with accessory genes that originated through HGT, we excluded genes that were classified as accessory genes by potential misclassification or gene deletion. Based on these criteria, 6393 (59.6 %) of the accessory genes were presumed to have been acquired by HGT. The majority (68.6 %) of inferred HGT genes were found to be related to genes found on different types of mobile genetic elements (Table S3).

### Large variation in retention of HGT genes over evolutionary time

Horizontally transferred genes acquired along a same branch that were physically adjacent to each other were grouped and considered to represent a single HGT event. A total of 2990 individual HGT events were identified. These HGT events were found to insert at a restricted number of genomic locations (249), with some core and tRNA genes acting as ‘hotspots’ for HGT (Table S4). The number of genes acquired per HGT event ranged from 1 to 37. Almost half of these events involved the acquisition of a single gene, and 90 % involved the acquisition of five genes or less. The number of HGT events along a single branch varied widely, even when correcting for branch length, and ranged from none to 230 (Fig. 1).

The highest number of HGT events was recorded along the branch leading to the ancestral node of a clade containing 56 strains (230 events, 364 genes). A high number of HGT events was also recorded along the branch departing from this node, leading to a sub-clade of 44 strains (87 events, 146 genes). An exceptional high number of HGT events was further found along the branch leading to the common ancestor of strains Carb01_63 and RIVM-EMC2982, two multidrug-resistant clinical strains isolated from different locations in the Netherlands (99 events, 188 genes). A total of 1165 HGT events, involving 4437 genes, were found to be strain-specific. Strain DHS01, a multidrug-resistant strain isolated in France, had the highest number of strain-specific HGT events (64 events involving 131 genes), although this number could be biassed by the absence of closely related strains in our dataset.

Each gene acquired through HGT was assigned a *residence time,* representing the evolutionary distance between the leaf on the phylogeny where a gene was found and the midpoint of the branch where this gene was acquired. A large number of genes acquired through HGT were characterized by a relatively short residence time, indicative of relatively recent transfers. The number of genes strongly decreased with increasing residence time, with only a small number of HGT genes found in the pan-genome of *P. aeruginosa* that were acquired early in its evolutionary history (Fig. S3).

### Codon usage of HGT genes converges towards the core genome over evolutionary time

Genes acquired by HGT had a much lower average % GC than core genes (58.8 % GC and 67.4 % GC, respectively), and a larger variation, with values ranging from 30.6 % GC to 73.3 % GC. The majority of HGT genes had a codon usage bias in the same direction as the core genes (88.0 % was characterized by a positive COUSIN value; Fig. 2). The strength of codon usage bias of these genes was however more variable and generally lower than that observed for the core genes (mean ENC=44.3±8.7 and 30.8±3.8, respectively; Fig. 2c). HGT genes with a codon usage bias in the opposite direction of the core genome (COUSIN <0) were generally also AT-biassed (average 44.8 % GC) and showed an overall lower codon usage bias strength (mean ENC=54.4±4.3).

The overall relationship between residence time and codon usage was determined using a non-parametric correlation. A linear regression model was used to examine the relation between codon usage and the minimal, maximal and median values for residence time intervals of 0.001. There was an overall positive correlation between a gene’s COUSIN value and its residence time (Kendall’s rank correlation: *τ*=0.027, *P*<0.05). This positive correlation is mainly driven by an overall increase of the minimal COUSIN values in function of residence time (linear model for minimal values: *P*<0.05; R^2^=0.93). The maximal COUSIN values showed a negative correlation with residence time (linear model for maximal values: *P*<0.05; R^2^=0.63). We did not find a significant correlation between the median COUSIN values and residence time (linear model for median values: *P*=0.1), indicating a converging trend of COUSIN values in function of residence time. The same trends were found for changes in CAI values in function of residence time (Fig. S4) and for changes in COUSIN values in function of residence time when transfers near the root or on branches with low bootstrap support (<70) were excluded.

### Gene amelioration does not systematically affect HGT genes

**Fig. 3.**
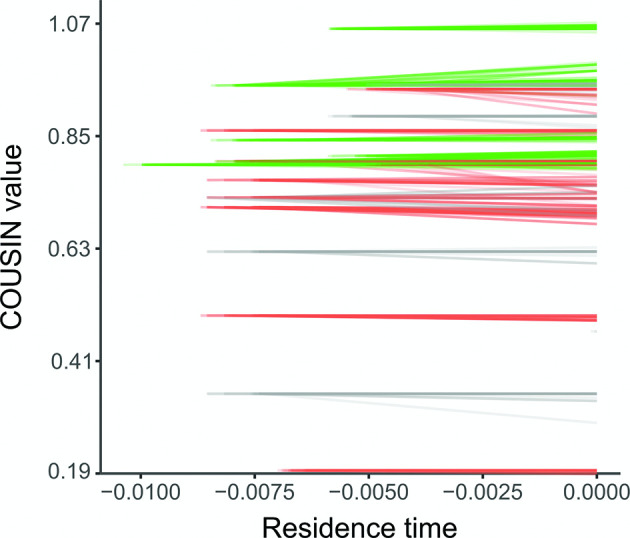
Changes in codon usage bias of HGT genes compared to their ancestral state. Each line represents the change in COUSIN value for a single CDS compared to its inferred ancestral sequence. Genes for which the mean change was positive are green, genes for which the mean change was negative are red and genes for which the change was non-significant are grey. Extant CDSs were given residence time 0 and the ancestral sequence was given the value of the residence time of its corresponding CDS.

We further assessed the potential importance of gene amelioration for changes in codon usage in function of residence time (i.e. are genes evolving towards the codon usage bias of the receiving genome through mutational processes?). To evaluate this hypothesis, we inferred the ancestral sequences for HGT genes that were represented by at least 20 or more extant sequences in our dataset. By applying this threshold we consequentially selected genes with the longest residence times, and thus those for which the strongest responses are expected. Codon usage bias in extant and ancestral sequences were then compared to evaluate changes due to mutational processes. This analysis showed variable responses for different genes (Fig. 3). Out of the 22 genes included in the analysis, six genes showed directional evolution towards the codon usage of the core genome, ten genes showed directional evolution opposite to the codon usage of the core genome, and six genes did not show any sign of directional evolution. When lowering the threshold to include HGT genes that were represented by at least ten or more extant sequences (*n*=78 genes), we found similar results: 20 genes showed directional evolution towards the codon usage of the core genome, 27 genes showed directional evolution opposite to the codon usage of the core genome, and 31 genes did not show any sign of directional evolution. Table 1 shows that directional changes in codon usage bias for genes with the longest residence time were generally caused by both synonymous and non-synonymous variation. However, most directional changes remained significant when only taking synonymous variation into account.

**Table 1.** Genes with significant directional changes in their codon usage bias compared to their inferred ancestral sequence

Gene	COUSIN_anc_	ΔCOUSIN_all_	ΔCOUSIN_syn_	*S*	*N*	No. of CDSs	Res. time
Bicarbonate transport system permease CmpB	0.949	+0.014	+0.014	12	3	61	0.007
Hypothetical protein	0.8	+0.009	+0.009	4	0	35	0.007
Alcohol dehydrogenase	0.842	+0.005	+0.005	8	3	46	0.007
Cyclic-di-GMP phosphodiesterase AdeB	0.811	+0.005	+0.005	6	7	26	0.005
Hypothetical protein	1.06	+0.003	n.s.	2	7	43	0.005
4-hydroxy-tetrahydrodipicolate synthase	0.794	+0.002	+0.005	7	5	31	0.009
Hypothetical protein	0.196	−0.001	−0.001	4	5	25	0.006
Hypothetical protein	0.861	−0.001	−0.001	3	0	31	0.008
Putative ribosomal N-acetyltransferase YdaF	0.801	−0.004	n.s.	3	5	37	0.007
HTH-type transcriptional regulator DmlR	0.73	−0.005	−0.008	6	5	23	0.008
Hypothetical protein	0.499	−0.005	−0.005	7	4	32	0.008
Hypothetical protein	0.794	−0.006	−0.007	3	1	36	0.004
Glycine cleavage system H protein	0.764	−0.008	−0.012	5	4	23	0.008
Putative manganese catalase	0.942	−0.008	−0.008	8	10	36	0.004
Glutathione S-transferase GST-6.0	0.711	−0.011	−0.009	5	4	23	0.008
Hypothetical protein	0.711	−0.016	−0.009	8	2	32	0.008

COUSIN_anc_.=COUSIN value of the ancestral sequence; ΔCOUSIN_all_=mean change in COUSIN values between ancestral and extant sequences. ΔCOUSIN_syn_=mean change in COUSIN values between ancestral and extant sequences when only synonymous variation is taken into account. *S*=number of positions with synonymous variation. *N*=number of positions with non-synonymous variation. No. of CDSs=number of CDSs in the analysis for each gene. Res. time=mean residence time for each gene.

### Increases in tRNA gene copy number are often associated with HGTs containing corresponding codons

After reconstructing evolutionary changes in tRNA gene copy numbers, a total of 98 events were detected involving a change in the tRNA gene content, with 83 of them being associated with the acquisition of genes through HGT. For most events this involved a change in the copy number of only a single tRNA gene (65 events), while some events showed variation in up to eight tRNA genes. Gene copy numbers of specific tRNA genes ranged from 0 to 8 copies, and 25 out of the 40 different tRNA genes found in *P. aeruginosa* had a variable gene copy number (Fig. S5). The tRNA^Lys^_TTT_ gene, which had either two, three or four copies, showed the highest rate of changes in gene copy number, with 26 individual gene duplications and 44 individual gene deletions. The number of observed changes in gene copy number for other variable tRNA genes ranged between 1 and 13. There were overall more cases where the gene copy number of a specific tRNA increased than decreased (98 and 75 cases, respectively). For tRNA^Ala^_CGC_, a gene that is generally absent in *P. aeruginosa*, four independent gains were recorded, indicative of multiple acquisitions through HGT.

If variation in tRNA gene content provided compensatory evolution in response to the acquisition of genes with a divergent codon usage, we expected to observe two patterns. First, on a codon level, we expected that an increment in the copy number of tRNA genes would be associated with HGT events increasing the frequency of codons that these tRNAs can translate. Second, on a gene level, we expected that changes in tRNA gene content should have a proportionally larger positive effect on the translation of recently acquired HGT genes than on other genes.

**Fig. 4.**
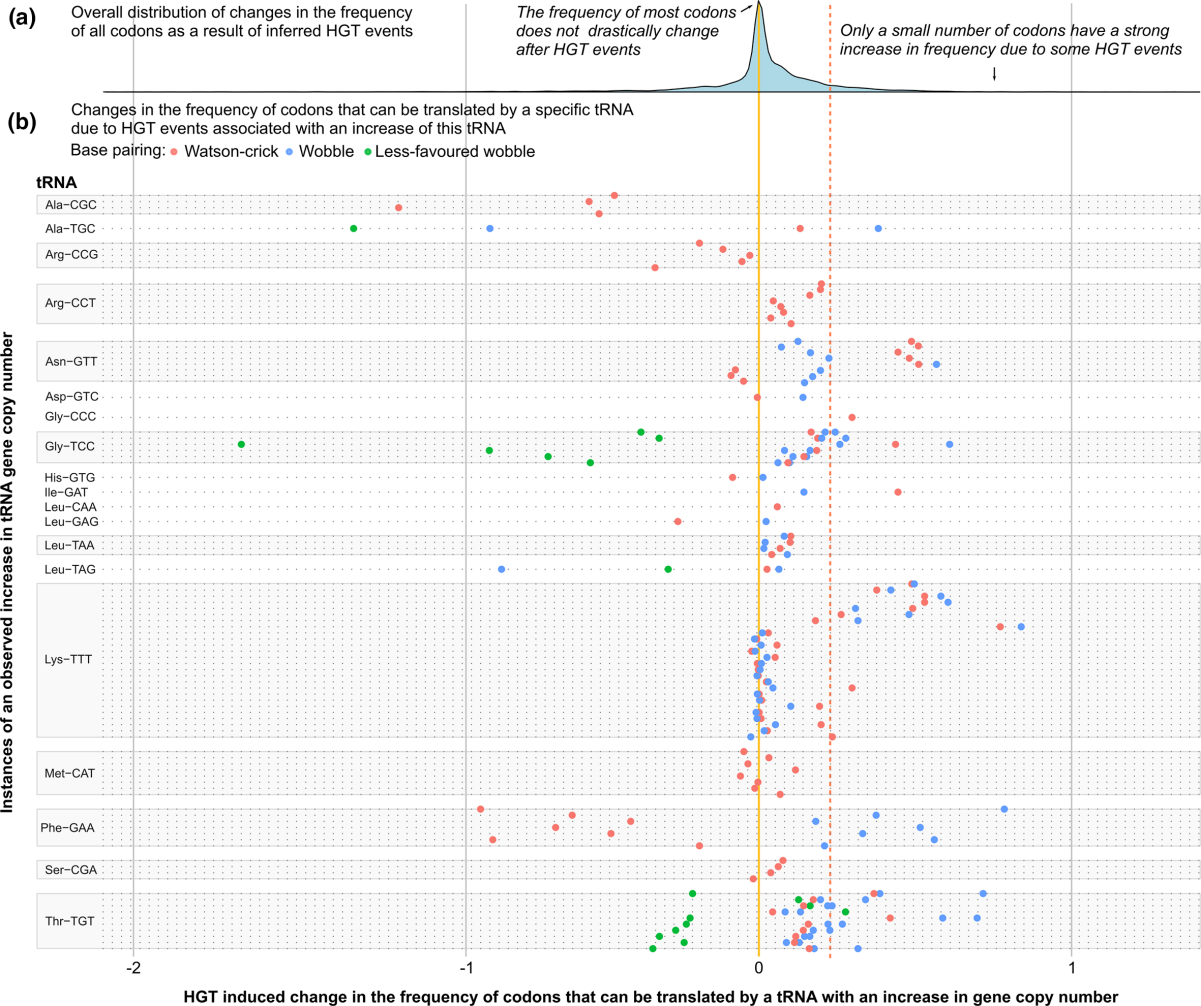
Changes in codon frequencies associated with increased tRNA gene copy numbers. Upper panel: distribution of changes in codon frequencies along branches due to HGT (expressed as changes in the expected number of a codon per 1000 bases). The yellow line indicates no change in codon frequency, the red line indicates the 90th percentile. Lower panel: each horizontal line represents a single event of increase in tRNA gene copy number and are grouped per tRNA gene; each point indicates a codon that was affected by this increase (red dots represent Watson–Crick base pairing, blue dots wobble base pairing and green dots less favoured wobble base pairing). The position along the horizontal axis indicates the change in codon frequency due to HGT along the branch where the increase in tRNA gene copy number was observed.

We performed a permutation test to evaluate if increments in tRNA gene copy number were associated with strong increases in the frequency of codons these tRNAs can translate. This analysis showed that there was a significant association with strong increases in the frequency of codons they can translate through Watson–Crick base pairing and wobble base pairing (*P* <0.05 for both permutation tests), but not for less favoured wobble base pairing interactions (Fig. 4). Although most increases in the copy number of tRNA genes were found to be associated with increases in the corresponding Watson–Crick and potential wobble codons, for some tRNA genes we observed opposite response patterns. Increases in the copy number of tRNA^Ala^_CGC_ and tRNA^Arg^_CCG_ genes were never associated with increases in their corresponding codons. Also, increases in tRNA^Phe^_GAA_ gene copy number were always associated with an increased frequency of its wobble codon (TTT), while its corresponding Watson–Crick codon (TTC) always decreased in frequency.

**Fig. 5.**
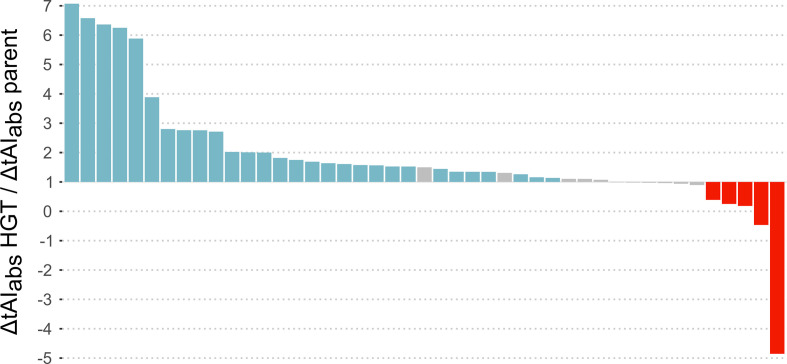
Change in ΔtAI_abs_ of horizontally transferred genes relative to the change in ΔtAI_abs_ of genes present at the parent node. Each bar represents an observed variation in tRNA gene content with an overall positive effect on translation. Values are obtained by dividing the expected change in translation efficiency (ΔtAI_abs_) for genes recently acquired through HGT and genes that were already present in each genome. Relative changes in ΔtAI_abs_ that were significantly larger for horizontally transferred genes are blue, those that were significantly larger for genes that were already present are red, non-significant differences are grey.

To analyse compensatory evolution on a gene level, we hypothesized that an expected increase in translation due to variation in tRNA gene content would be proportionally larger for recently acquired HGT genes than for other genes. In our dataset, there were 45 changes in tRNA gene content with an expected overall positive effect on translation, as determined by a mean positive ΔtAI_abs_ of all genes affected by this change. Of these, 29 changes in tRNA gene content caused a proportionally larger ΔtAI_abs_ for HGT genes than for genes that were already present at the parent node (Mann–Whitney U-test for each case: *P*<0.05; Fig. 5). In only five cases was the ΔtAI_abs_ higher for genes present at the parent node than for HGT genes (Mann–Whitney U-test for each case: *P*<0.05), while in the remaining 11 cases there was no difference between the ΔtAI_abs_ of HGT genes and genes present at the parent node (Fig. 5). On average, the increase in ΔtAI_abs_ was 2.1 times higher for HGT genes than for genes present at the parent node.

## Discussion

Evolutionary responses related to codon usage of horizontally transferred genes were investigated in *P. aeruginosa* using a phylogeny-based approach. We showed that there was selection on codon usage of genes acquired through HGT, with their codon usage converging toward that of the core genome over evolutionary time. Gene amelioration, through the accumulation of synonymous mutations after HGT, did not seem to be driving the changes in codon usage towards that of the receiving organism. This favours the hypothesis that long-term retention of horizontally transferred genes was strongly influenced by their initial codon usage. We further found evidence that compensatory evolution might be important for the integration of horizontally transferred genes, as variation in tRNA gene content was often associated with the acquisition of genes for which the observed variation in tRNA gene content could enhance their expression.

Previous work already indicated that for multiple bacterial species the likelihood of successful HGT increases when the codon usage of transferred genes is similar to that of the receiving organism [11, 40]. Our results show that this also plays a role in *P. aeruginosa*, and additionally shows a role for codon usage in determining the probability of long-term gene retention. Initially there seems to be a relatively weak selection against the uptake of genes with an atypical codon usage. *P. aeruginosa* was found to incorporate genes exhibiting a wide range of codon usage, including unbiased genes and genes with a bias opposite to that of the core genes. However, the majority of recently transferred genes still had a codon usage bias in the same direction as the core genes, but generally less pronounced. Over evolutionary time, we observed selection against genes with a codon usage bias opposite to that of the core genes or with a very strong bias in the same direction as the core genes.

Two different mechanisms could drive the observed change in overall codon usage of genes acquired through HGT over evolutionary time. First, loss rates of genes with an atypical codon usage could be higher than for genes with a codon usage similar to that of the receiving organism. Especially if the maintenance and expression of genes with an atypical codon usage is costly, they are expected to be purged by natural selection [2]⁠. Even if the transferred genes do not confer a fitness cost, but are neither able to provide a net fitness benefit to the receiving organism due to their atypical codon usage, they will be prone to loss by genetic drift [41]⁠. Second, gene amelioration due to the local mutational bias and/or selection on synonymous mutations could drive the codon usage of a transferred gene towards that of the receiving organism. We evaluated the potential role of gene amelioration and found no clear indications that synonymous mutations in horizontally transferred genes are causing the observed changes in codon usage. Medrano-Soto *et al*. similarly argued against a role for amelioration after horizontal gene transfer [11]⁠. However, their argument was based on a strong initial selection on codon usage after HGT, removing opportunities for amelioration. Our analysis shows that even when genes with a deviating codon usage are retained for a long time after a HGT event, amelioration responses can be absent. Although amelioration is assumed to equally affect all synonymous positions of non-preferred codons [13]⁠, it must be noted that with our approach parallel evolution (i.e. the occurrence of same synonymous mutations in different lineages) could cause an underestimation of the importance of this mechanism.

Amelioration is expected to occur when transferred genes are exposed to a new mutational and/or selective regime present in the receiving organism [13]⁠. The codon usage of the core genes is on the other hand assumed to be in equilibrium with these forces. There has been some speculation on the processes shaping the overall high GC-content and strong codon usage bias in the *P. aeruginosa* core genes. Dettman *et al*. experimentally showed that *P. aeruginosa* has a mutational bias towards AT [42]. The same study noted that mismatch-repair (MMR) deficient strains had an inverse bias towards GC, and they hypothesized that a high prevalence of MMR deficient strains in combination with recombination would lead to a high equilibrium GC content. Furthermore, other mechanisms such as GC-biassed gene conversion [43]⁠ or selection for high GC content [44]⁠ could also play a role. The processes that are driving the high GC content and codon usage of the core genes did not seem to systematically induce synonymous variation in the same direction in horizontally transferred genes. Given that our data points towards the absence of amelioration in *P. aeruginosa*, we infer that the observed convergence in codon usage of HGT genes towards that of the core genes was mainly driven by selective gene retention based on their initial codon usage.

Although there was a general pattern of selective retention of HGT genes based on their degree of similarity in codon usage to core genes, there were still several horizontally transferred genes with a long residence time that maintained an atypical codon usage bias. A notable example was the LESGI-7 genomic island (Fig. 2: residence time ~0.008 and COUSIN <0; described in [45])⁠, which was inferred to be an ancient transfer at the base of the large clade containing strain PA14, and is shared by a large number of ecologically diverse strains. The ten genes on this genomic island are involved in determining the lipopolysaccharide O-antigen serotype and exhibit a codon usage bias opposite to that of the core genes. A possible reason for the conservation of an atypical codon usage could be that gene expression is under the control of xenogeneic silencers that target DNA segments with a higher % AT than the chromosomal average [46]⁠. There are indications that xenogeneic silencing can act as a buffer against negative fitness consequences of HGT [47]⁠ and can facilitate the integration of horizontally acquired genes into existing regulatory networks [48]⁠. In the *P. aeruginosa* strain PAO1, the xenogeneic silencer *MvaT* and its paralogue *MvaU* are known to be critical for regulating the expression of virulence-associated and prophage genes [49]. It is highly probable that genes on LESGI-7, because of their high AT content, are targeted by the same xenogeneic silencing proteins. If regulation of genes on LESGI-7 is dependent on the interaction with these proteins, it would impose selection for maintaining a high AT-content to preserve regulation of expression and cause the conservation of their atypical codon usage.

We further found strong indications that the observed variation in tRNA gene content in *P. aeruginosa* was – at least partially – accommodating for the expression of horizontally transferred genes. Similar indications were found in *E. coli* using a comparative genomics approach [18]⁠. In this species it has also been shown, through the experimental manipulation of tRNA gene copy number, that this mechanism can provide an efficient response to enhance the expression of genes with a codon usage that is not adapted to the tRNA pool [50]⁠. Our results indicate that this mechanism might be more widespread in other bacterial species as a means of providing compensatory evolution related to HGT. Although we often found a strong association between variation in tRNA gene content and HGT events, there were also a large number of HGT events that caused the influx of atypical codons but were not associated with variation in tRNA gene content. Varying tRNA gene copy numbers is only one of several possibilities to compensate for the inefficient expression of genes with an atypical codon usage. Mechanisms such as the regulation of the expression of specific tRNA genes [51]⁠, the action of tRNA modifying enzymes that can extend wobble base pairing [52] or mutations in horizontally transferred genes that increase their expression [53, 54]⁠ are other possibilities to improve the expression of horizontally transferred genes in their new host genome, but our analysis did not allow to test for these mechanisms. We also observed an increase in tRNA gene copy numbers without an associated increase in the frequency of codons that can be translated by these tRNAs. This indicates that not all variation in tRNA gene copy numbers is due to HGT related compensatory evolution, but it could either be explained by neutral variation or by adaptive responses to other changes such as an increased expression of genes containing corresponding codons.

Our approach did not allow to discriminate between different mechanisms causing variation in tRNA gene content. To obtain a more detailed image of compensatory evolution in *P. aeruginosa,* it will be necessary to analyse the contribution of genomic tRNA genes duplications, tRNA gene acquisition through HGT and mutations in the anticodons of tRNA genes [55]⁠ to tRNA gene content variation. For example, it would be interesting to know if there are cases of protein coding genes that have been transferred along with tRNA genes that could enhance their expression, similar to what has been observed in viruses [56]⁠.

Our study shows that codon usage plays an important role in shaping the repertoire of horizontally transferred genes in the *P. aeruginosa* accessory genome and that the incorporation of genes with an atypical codon usage might elicit changes elsewhere in the genome to enhance their integration. The results presented here are however correlative, and there is a need for experiments that quantify the selection coefficient on different synonymous versions of horizontally transferred genes. Experimental manipulation of tRNA gene content will also allow estimation of the fitness effects of horizontally transferred genes in different genomic contexts. Increasing our knowledge on the importance of, and potential evolutionary responses to codon usage in the context of HGT will allow for a better assessment of the potential for the emergence of antibiotic resistance and increased virulence in pathogenic bacteria through HGT.

## Supplementary Data

Supplementary material 1

## References

[1] Vos M, Hesselman MC, Te Beek TA, van Passel MWJ, Eyre-Walker A (2015). Rates of lateral gene transfer in Prokaryotes: high but why?. Trends Microbiol.

[2] Koskiniemi S, Sun S, Berg OG, Andersson DI (2012). Selection-driven gene loss in bacteria. PLoS Genet.

[3] Baltrus DA (2013). Exploring the costs of horizontal gene transfer. Trends Ecol Evol.

[4] Grantham R, Gautier C, Gouy M, Mercier R, Pavé A (1980). Codon catalog usage and the genome hypothesis. Nucleic Acids Res.

[5] Novoa EM, Jungreis I, Jaillon O, Kellis M (2019). Elucidation of codon usage signatures across the domains of life. Mol Biol Evol.

[6] Hershberg R, Petrov DA (2008). Selection on Codon Bias. Annu Rev Genet.

[7] Rocha EPC (2004). Codon usage bias from tRNA’s point of view: Redundancy, specialization, and efficient decoding for translation optimization. Genome Res.

[8] Higgs PG, Ran W (2008). Coevolution of codon usage and trna genes leads to alternative stable states of biased codon usage. Mol Biol Evol.

[9] Plotkin JB, Kudla G (2011). Synonymous but not the same: the causes and consequences of codon bias. Nat Rev Genet.

[10] Presnyak V, Alhusaini N, Chen Y-H, Martin S, Morris N (2015). Codon optimality is a major determinant of mrna stability. Cell.

[11] Medrano-Soto A, Moreno-Hagelsieb G, Vinuesa P, Christen JA, Collado-Vides J (2004). Successful lateral transfer requires codon usage compatibility between foreign genes and recipient genomes. Mol Biol Evol.

[12] Tuller T, Girshovich Y, Sella Y, Kreimer A, Freilich S (2011). Association between translation efficiency and horizontal gene transfer within microbial communities. Nucleic Acids Res.

[13] Lawrence JG, Ochman H (1997). Amelioration of bacterial genomes: Rates of change and exchange. J Mol Evol.

[14] Bedhomme S, Amorós-Moya D, Valero LM, Bonifaci N, M-À P (2019). Evolutionary changes after translational challenges imposed by horizontal gene transfer. Genome Biol Evol.

[15] Harrison E, Dytham C, Hall JPJ, Guymer D, Spiers AJ (2016). Rapid compensatory evolution promotes the survival of conjugative plasmids. Mob Genet Elements.

[16] Tuller T (2011). Codon bias, tRNA pools, and horizontal gene transfer. Mob Genet Elements.

[17] Dong H, Nilsson L, Kurland CG (1996). Co-variation of trna abundance and codon usage in *Escherichia coli* at different growth rates. J Mol Biol.

[18] McDonald MJ, Chou C-H, Swamy KBS, Huang H-D, Leu J-Y (2015). The evolutionary dynamics of trna-gene copy number and codon-use in *E. Coli*. BMC Evol Biol.

[19] Harrison EM, Carter MEK, Luck S, Ou H-Y, He X (2010). Pathogenicity islands PAPI-1 and PAPI-2 contribute individually and synergistically to the virulence of Pseudomonas aeruginosa strain PA14. Infect Immun.

[20] Kung VL, Ozer EA, Hauser AR (2010). The accessory genome of *Pseudomonas aeruginosa*. Microbiol Mol Biol Rev.

[21] Roy PH, Tetu SG, Larouche A, Elbourne L, Tremblay S (2010). Complete genome sequence of the multiresistant taxonomic outlier pseudomonas aeruginosa PA7. PLoS One.

[22] Seemann T (2014). Prokka: rapid prokaryotic genome annotation. Bioinformatics.

[23] Chan PP, Lin BY, Mak AJ, Lowe TM (2019). Trnascan-se 2.0: Improved detection and functional classification of transfer RNA genes. bioRxiv.

[24] Miele V, Penel S, Duret L (2011). Ultra-fast sequence clustering from similarity networks with SiLiX. BMC Bioinformatics.

[25] Page AJ, Cummins CA, Hunt M, Wong VK, Reuter S (2015). Roary: rapid large-scale prokaryote pan genome analysis. Bioinformatics.

[26] Freschi L, Vincent AT, Jeukens J, Emond-Rheault J-G, Kukavica-Ibrulj I (2019). The *Pseudomonas aeruginosa* Pan-genome provides new insights on its population structure, horizontal gene transfer, and pathogenicity. Genome Biol Evol.

[27] Katoh K, Standley DM (2013). MAFFT multiple sequence alignment software version 7: Improvements in performance and usability. Mol Biol Evol.

[28] Nguyen LT, Schmidt HA, von Haeseler A, Minh BQ (2015). IQ-TREE: A fast and effective stochastic algorithm for estimating maximum-likelihood phylogenies. Mol Biol Evol.

[29] Kalyaanamoorthy S, Minh BQ, Wong TKF, von Haeseler A, Jermiin LS (2017). ModelFinder: fast model selection for accurate phylogenetic estimates. Nat Methods.

[30] Berger SA, Krompass D, Stamatakis A (2011). Performance, accuracy, and web server for evolutionary placement of short sequence reads under maximum likelihood. Syst Biol.

[31] Tria FDK, Landan G, Dagan T (2017). Phylogenetic rooting using minimal ancestor deviation. Nat Ecol Evol.

[32] Boussau B, Scornavacca C (2020). Reconciling Gene Trees with Species Trees (Internet).

[33] Csűös M (2010). Count: evolutionary analysis of phylogenetic profiles with parsimony and likelihood. Bioinformatics.

[34] Bourret J, Alizon S, Bravo IG (2019). COUSIN (Codon Usage Similarity Index): A normalized measure of Codon usage preferences. Genome Biol Evol.

[35] Sharp PM, Li WH (1986). An evolutionary perspective on synonymous codon usage in unicellular organisms. J Mol Evol.

[36] Wright F (1990). The ‘effective number of codons’ used in a gene. Gene.

[37] Murphy F, Ramakrishnan V (2004). Structure of a purine-purine wobble base pair in the decoding center of the ribosome. Nat Struct Mol Biol.

[38] dos Reis M, Savva R, Wernisch L (2004). Solving the riddle of codon usage preferences: a test for translational selection. Nucleic Acids Res.

[39] Sabi R, Volvovitch Daniel R, Tuller T (2016). stAI calc: tRNA adaptation index calculator based on species-specific weights. Bioinformatics.

[40] Bolotin E, Hershberg R (2017). Horizontally acquired genes are often shared between closely related bacterial species. Front Microbiol.

[41] Kuo CH, Moran NA, Ochman H (2009). The consequences of genetic drift for bacterial genome complexity. Genome Res.

[42] Dettman JR, Sztepanacz JL, Kassen R (2016). The properties of spontaneous mutations in the opportunistic pathogen *Pseudomonas aeruginosa*. BMC Genomics.

[43] Lassalle F, Périan S, Bataillon T, Nesme X, Duret L (2015). GC-Content evolution in bacterial genomes: the biased gene conversion hypothesis expands. PLoS Genet.

[44] Hildebrand F, Meyer A, Eyre-Walker A (2010). Evidence of selection upon genomic gc-content in bacteria. PLoS Genet.

[45] Jani M, Mathee K, Azad RK (2016). Identification of novel genomic islands in Liverpool epidemic strain of *Pseudomonas aeruginosa* using segmentation and clustering. Front Microbiol.

[46] Singh K, Milstein JN, Navarre WW (2016). Xenogeneic silencing and its impact on bacterial genomes. Annu Rev Microbiol.

[47] Ali SS, Soo J, Rao C, Leung AS, Ngai DH-M (2014). Silencing by H-NS potentiated the evolution of salmonella. PLoS Pathog.

[48] Will WR, Navarre WW, Fang FC (2015). Integrated Circuits: How Transcriptional Silencing and Counter-Silencing Facilitate Bacterial Evolution.

[49] Castang S, Dove SL (2012). Basis for the essentiality of H-NS family members in Pseudomonas aeruginosa. J Bacteriol.

[50] Du M-Z, Wei W, Qin L, Liu S, Zhang A-Y (2017). Co-adaption of tRNA gene copy number and amino acid usage influences translation rates in three life domains. DNA Res.

[51] Torrent M, Chalancon G, De Groot NS, Wuster A, Madan Babu M (2018). Cells alter their tRNA abundance to selectively regulate protein synthesis during stress conditions. Sci Signal.

[52] Endres L, Dedon PC, Begley TJ (2015). Codon-biased translation can be regulated by wobble-base tRNA modification systems during cellular stress responses. RNA Biol.

[53] Amorós-Moya D, Bedhomme S, Hermann M, Bravo IG (2010). Evolution in regulatory regions rapidly compensates the cost of nonoptimal codon usage. Mol Biol Evol.

[54] Bedhomme S, Perez Pantoja D, Bravo IG (2017). Plasmid and clonal interference during post horizontal gene transfer evolution. Mol Ecol.

[55] Yona AH, Bloom-Ackermann Z, Frumkin I, Hanson-Smith V, Charpak-Amikam Y (2013). Trna genes rapidly change in evolution to meet novel translational demands. Elife.

[56] Limor-Waisberg K, Carmi A, Scherz A, Pilpel Y, Furman I (2011). Specialization versus adaptation: two strategies employed by cyanophages to enhance their translation efficiencies. Nucleic Acids Res.

