# Supplementary material for: Evolutionary responses to codon usage of horizontally transferred genes in Pseudomonas aeruginosa: gene retention, amelioration and compensatory evolution
Source: Microb Genom. 2021 Jun 24;7(6):000587. doi: 10.1099/mgen.0.000587 (PMC8461475; doi:10.1099/mgen.0.000587)
Supplement: Supplementary material 1 [file mgen-7-0587-s001.pdf]

Figure S1

Schematic overview of the bioinformatic pipeline used in this study. A: pan-genome analysis; B. strain phylogeny; C. Filter accessory genes. D. Ancestral state reconstruction and inference of HGT's

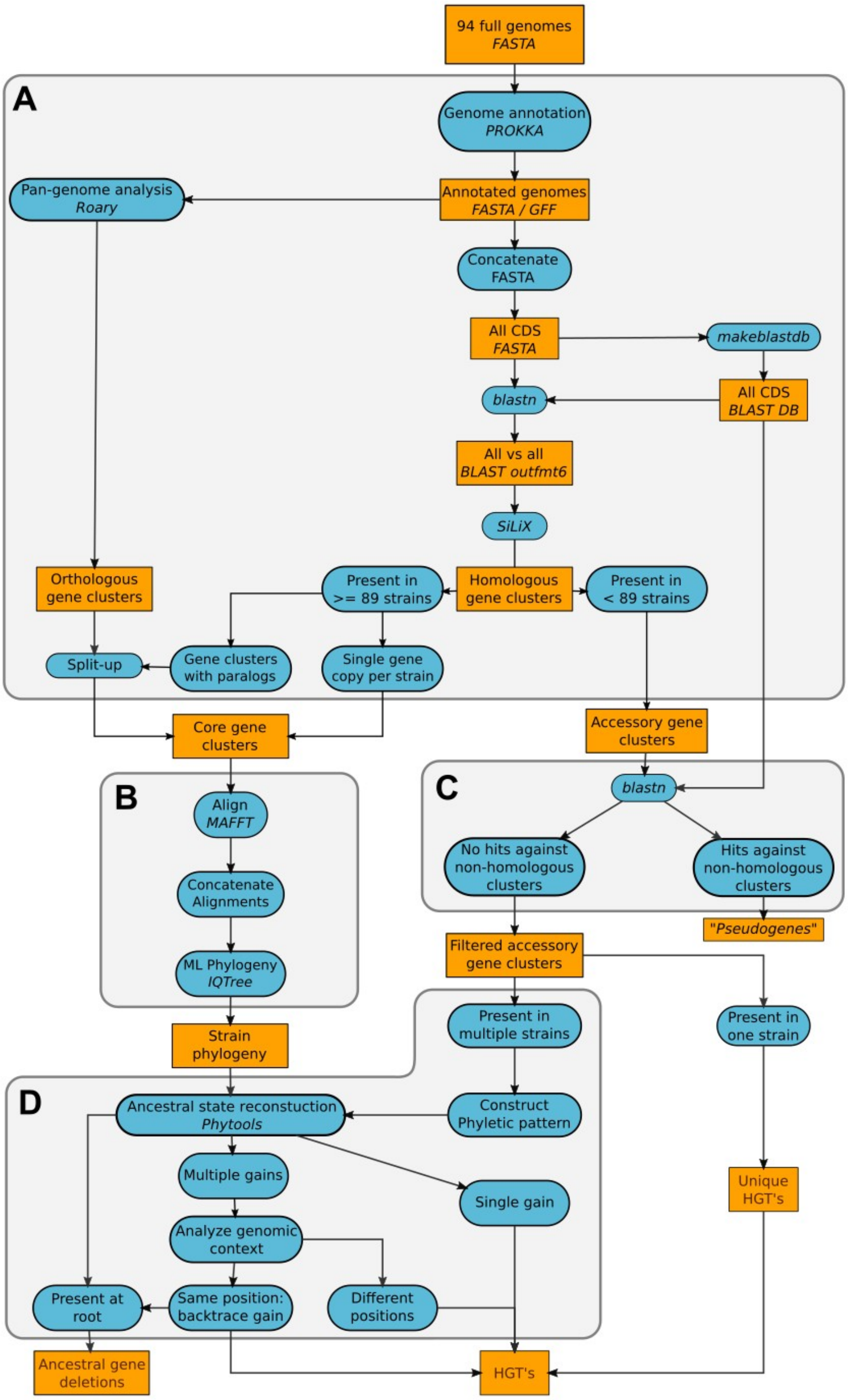

Figure S2

Phylogenetic tree containing the *P. aeruginosa* strains used in this study. The tree was produced using the concatenated alignment of all the core genes and inferred using a GTR+F+R9 model of substitution. The tree was rooted at midpoint. Branch support was obtained with 1000 bootstrap replicates. Bootstrap values are indicated on the nodes.

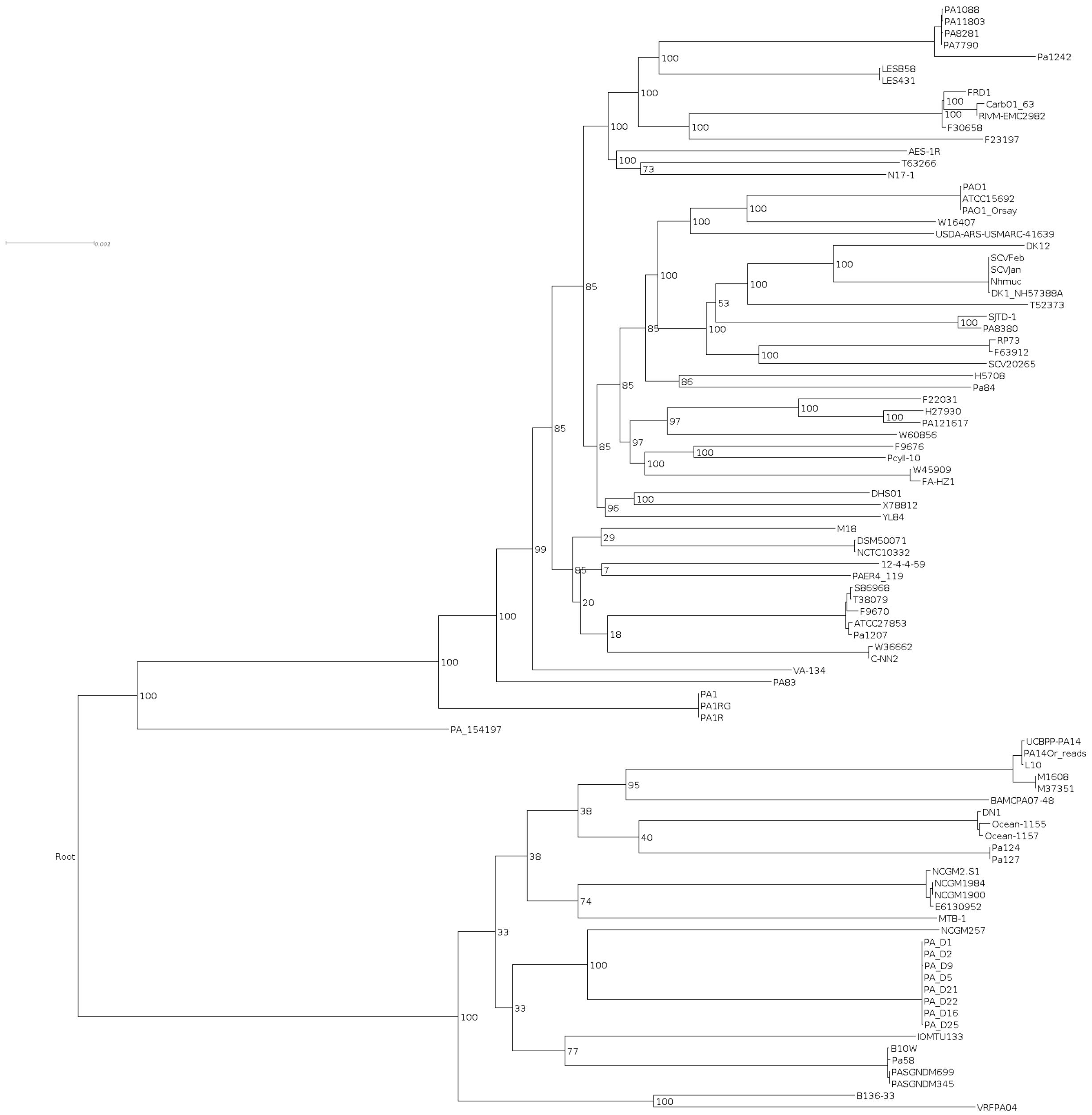

Figure S3

Distribution of the residence time of genes acquired by HGT. The largest fraction of genes were recently acquired, while at longer residence times the amount of genes was strongly reduced. The inset shows the distribution of distances between the tree tips and all internal nodes of the strain phylogeny, indicating that the gaps in the distribution of residence time are potentially caused by the structure of the strain phylogeny.

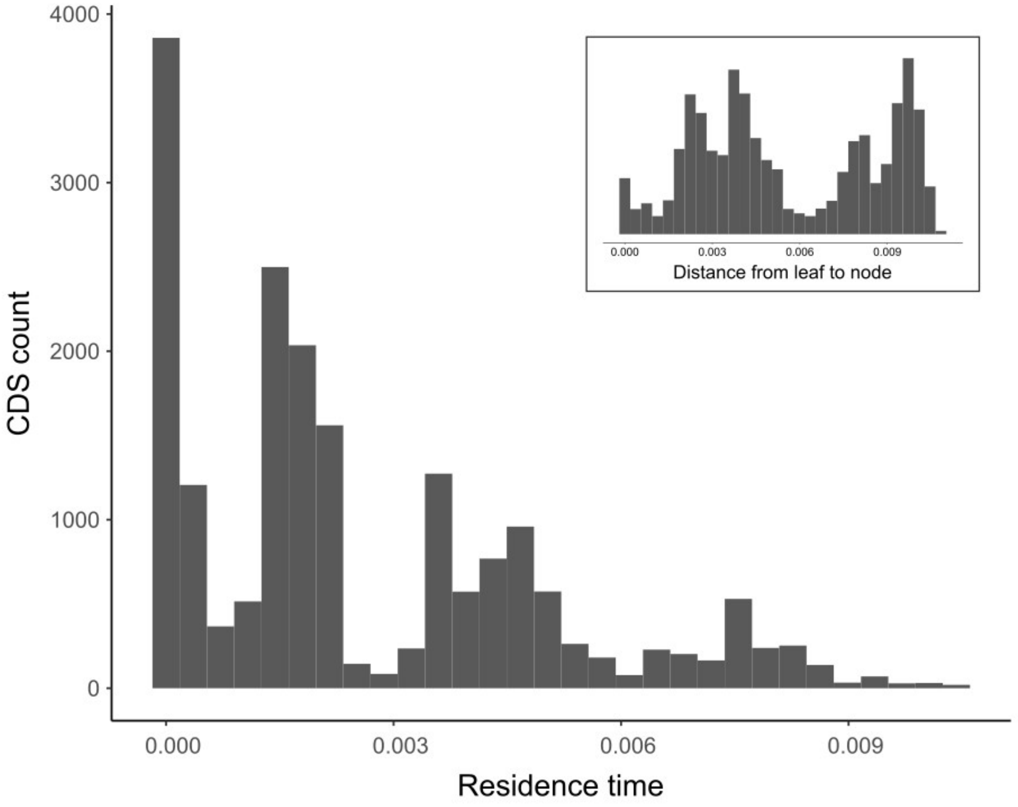

Figure S4

Codon usage bias of HGT genes in relation to their residence time. Scatterplot of the CAI value of HGT genes in function of their residence time. The blue and red regression lines were determined by taking respectively the minimal and maximal CAI value for each residence time interval of 0.001.

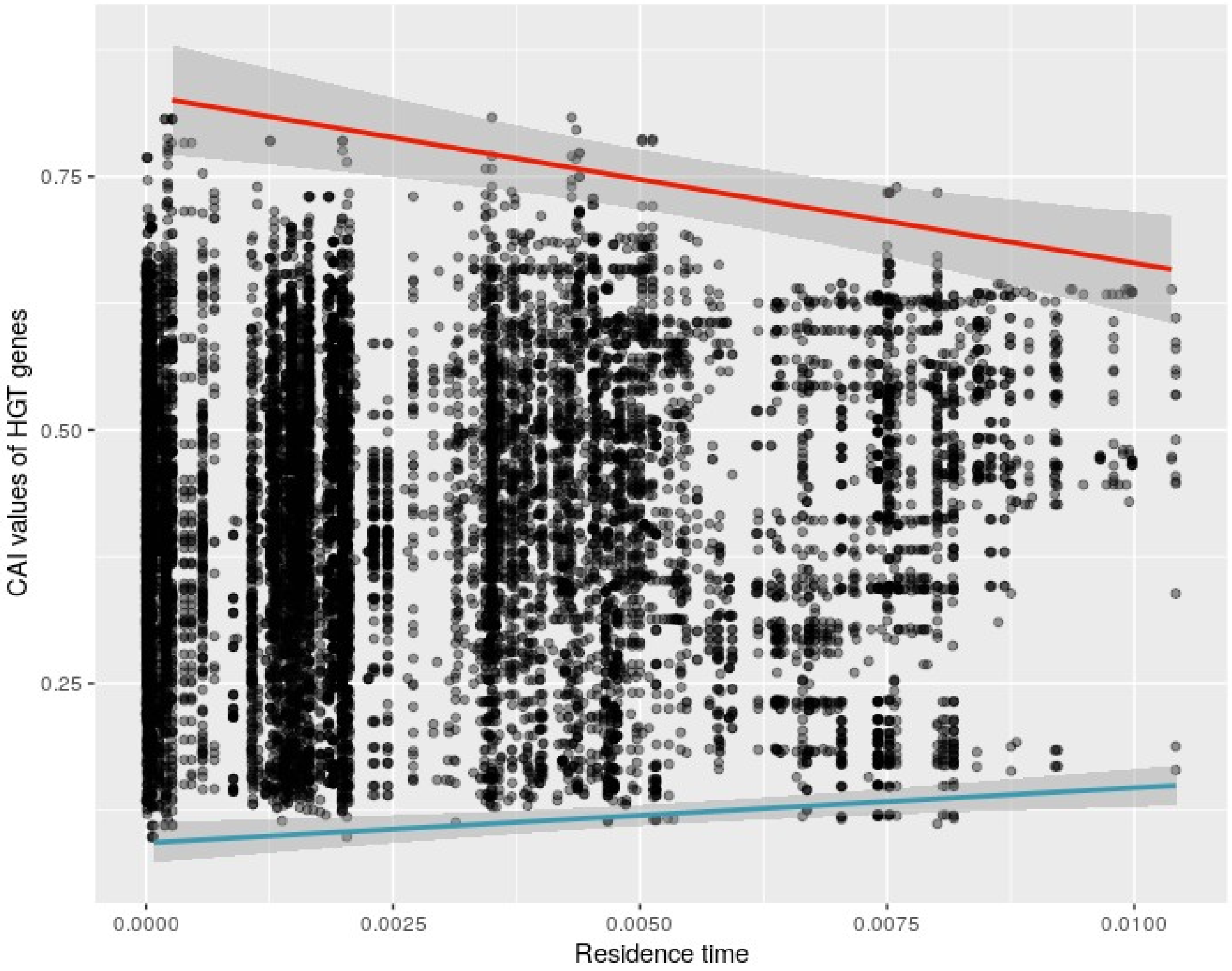

Figure S5

Variation in tRNA gene copy number in *P. aeruginosa*. Each tRNA gene is specified by its anticodon, isoacceptor tRNA's are grouped by amino acid. The y-axis indicates the copy number that was observed for a specific tRNA gene, the circle size indicates the relative fraction of strains having a specific tRNA gene copy number.

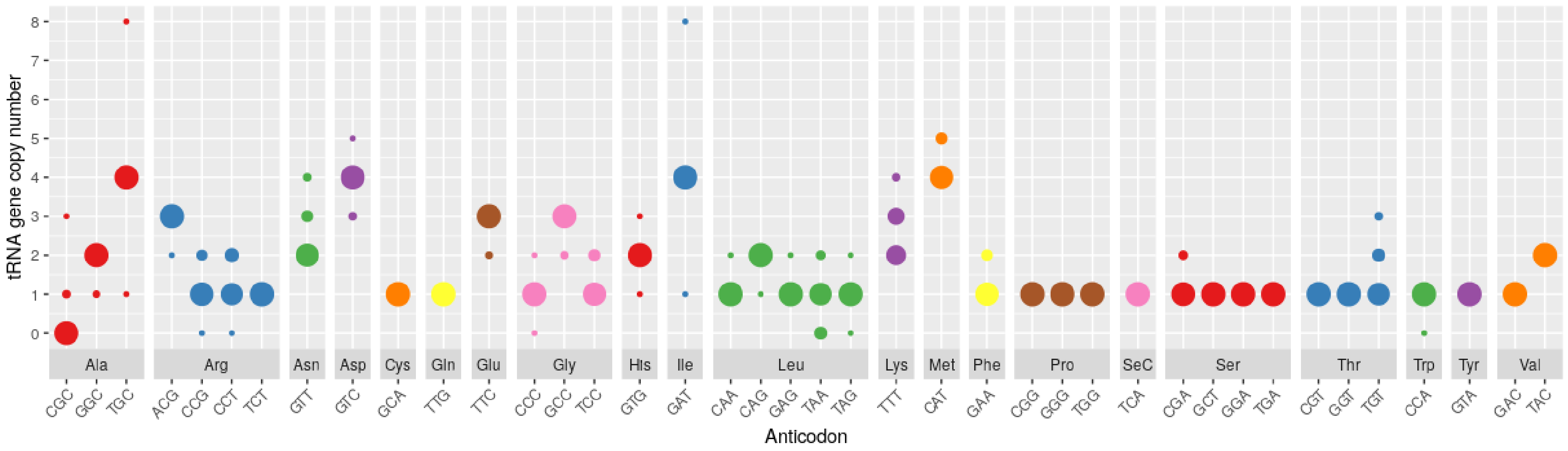

Table S1: *P. aeruginosa* genome data used in this study

Strain_name	RefSeq_accession	Environment	Comment	Genome_size(bp)	CDS_ncbi	CDS_prokka
PAO1	GCF_000006765	Laboratory	Type strain	6264404	5571	5671
UCBPP-PA14	GCF_000014625	Clinical		6537648	5892	5901
LESB58	GCF_000026645	Clinical		6601757	5931	6048
AES-1R	GCF_000220025	Clinical		6267620	na	5839
M18	GCF_000226155	Rhizosphere		6327754	5684	5758
DK12	GCF_000271365	Clinical		6402658	5884	5855
SJTD-1	GCF_000271985	Soil		6243825	5603	5637
NCGM2.S1	GCF_000284555	Clinical		6764661	6269	6214
B136-33	GCF_000359505	Clinical		6421010	5828	5811
RP73	GCF_000414035	Clinical		6342034	5762	5791
VRFPA04	GCF_000473745	Clinical		6818030	5778	6128
DHS01	GCF_000496455	Clinical		7055752	6524	6605
PA1	GCF_000496605	Clinical		6498072	5902	5938
PA1R	GCF_000496645	Clinical		6309305	5612	5776
MTB-1	GCF_000504045	Soil		6580038	6078	5991
LES431	GCF_000508765	Clinical		6550070	5964	5995
SCV20265	GCF_000510305	Clinical		6725183	6299	6175
YL84	GCF_000524595	Soil	Compost	6433441	5821	5843
F22031	GCF_000816985	Clinical		6603099	6077	6014
NCGM1984	GCF_000829255	Clinical		6850954	6291	6299
NCGM1900	GCF_000829275	Clinical		6814936	6358	6299
FRD1	GCF_000829885	Clinical		6712339	5852	6184
Carb01_63	GCF_000981825	Clinical		7497593	6900	7010
DSM50071	GCF_001045685	Laboratory	Type strain	6317050	5710	5730
F9676	GCF_001077475	Plant	From diseased rice s	6368008	5721	5759
PA1RG	GCF_001293085	Clinical	Bacteriophage resista	6500439	5910	5943
VA-134	GCF_001447845	Clinical	Isolated from burn pa	6400418	5725	5799
NCTC10332	GCF_001457615	Laboratory	Type strain	6316979	5778	5773
12-4-4(59)	GCF_001482325	Clinical	Blood culture of a bur	6431911	5802	5850
S86968	GCF_001515845	Clinical	From cancer patient	6934277	6436	6348
T38079	GCF_001515915	Clinical	From cancer patient	6795741	6215	6205
T52373	GCF_001516005	Clinical	From cancer patient	6322459	5720	5734
T63266	GCF_001516105	Clinical	From cancer patient	6456866	5952	5907
W16407	GCF_001516165	Clinical	From cancer patient	6808844	6262	6254
W36662	GCF_001516185	Clinical	From cancer patient	6791731	6291	6186
W45909	GCF_001516205	Clinical	From cancer patient	6777566	6322	6214
W60856	GCF_001516225	Clinical	From cancer patient	6896700	6397	6304
F23197	GCF_001516245	Clinical	From cancer patient	6517340	5968	5890
F30658	GCF_001516265	Clinical	From cancer patient	7273258	6760	6695
H5708	GCF_001516305	Clinical	From cancer patient	6334378	5849	5731
H27930	GCF_001516325	Clinical	From cancer patient	6568228	6003	5948
M1608	GCF_001516365	Clinical	From cancer patient	6460023	5940	5883
M37351	GCF_001516385	Clinical	From cancer patient	6897231	6390	6349
USDA-ARS-USMAR	GCF_001518975	Mammal	Cow with bovine resp	6364583	5775	5760
X78812	GCF_001542795	Clinical	From cancer patient	6348761	5836	5729
F9670	GCF_001542835	Clinical	From cancer patient	6794354	6071	6266
NCGM257	GCF_001547955	Clinical	Urinary infection	7090694	6892	6618
PA8380	GCF_001548135	Clinical	Human gut	6613260	6198	6047
IOMTU133	GCF_001548335	Clinical	Multidrug resistant	6897018	6433	6281
F63912	GCF_001594325	Clinical	From cancer patient	6618768	6211	6099
N17-1	GCF_001606045	Soil	Aflatoxin degrading st	6370730	5763	5801
ATCC27853	GCF_001618925	Clinical	Nosocomial infection	6827737	6239	6287
BAMCPA07-48	GCF_001632245	Clinical	Combat injury wound	7021552	6355	6533
PA121617	GCF_001679685	Clinical	Sputum from respirat	6853510	6225	6280
PA_D1	GCF_001721745	Clinical	Sputum from ventilato	6643823	6017	6033
PA_D2	GCF_001721765	Clinical	Sputum from ventilato	6642996	6017	6030
PA_D9	GCF_001721785	Clinical	Sputum from ventilato	6645477	6014	6040
PA_D16	GCF_001721805	Clinical	Sputum from ventilato	6681975	6042	6070
PA_D22	GCF_001721825	Clinical	Sputum from ventilato	6681981	6037	6072
PA_D25	GCF_001721845	Clinical	Sputum from ventilato	6683204	6036	6080
DN1	GCF_001722005	Soil		6959251	6024	6470
PA_D5	GCF_001722025	Clinical	Sputum from ventilato	6681992	6042	6065
PA_D21	GCF_001722045	Clinical	Sputum from ventilato	6639108	6012	6031
ATCC15692	GCF_001729505	Clinical	Infected wound	6276434	5667	5693
FA-HZ1	GCF_001750705	Wastewater	DBF degrading strain	6866790	6064	6439
PA1088	GCF_001792835	Clinical	Urine	6721480	6199	6160
PA8281	GCF_001792855	Clinical	Tracheal aspirate	6928736	6426	6392
PA11803	GCF_001792875	Clinical	Bloodstream	7006578	6582	6562
PA7790	GCF_001870265	Clinical	Tracheal aspirate	7067711	6600	6577
B10W	GCF_001874465	Wastewater		6723378	6069	6168

PAER4_119	GCF_001879525	Laboratory	Lindberg collection	6504659	5881	5881
SCVFeb	GCF_001900195	Mammal	Murine model	6213026	5619	5627
SCVJan	GCF_001900225	Mammal	Murine model	6213029	5619	5627
Nhmuc	GCF_001900265	Clinical		6213276	5619	5628
PA_154197	GCF_002075065	Clinical		6445239	5863	5856
RIVM-EMC2982	GCF_002085605	Clinical		7380063	6762	6873
E6130952	GCF_002085755	Clinical	Respiratory failure	7077406	6586	6592
PASGNDM699	GCF_002104595	Clinical	NDM-1 producing stra	6985102	6428	6426
PASGNDM345	GCF_002104615	Clinical	NDM-1 producing stra	6893164	6316	6328
Pa124	GCF_002192475	Clinical	Ventilator-associate	7008516	6369	6385
Pa58	GCF_002192495	Clinical	Ventilator-associate	7241575	6564	6730
Pa84	GCF_002205335	Clinical	Ventilator-associate	6566724	5964	6034
Pa127	GCF_002205355	Clinical	Ventilator-associate	7148302	6459	6574
Pa1242	GCF_002205375	Clinical	Blood	7050510	6212	6584
Pa1207	GCF_002208645	Clinical	Blood	7411863	6721	6905
PA83	GCF_002215345	Clinical	Blood	7214314	6610	6622
L10	GCF_002223805	Marine	Halobiotic reed	6661962	6137	6018
Ocean-1155	GCF_002237405	Marine	Open ocean, Pacific	6952237	6361	6398
Ocean-1157	GCF_002237425	Marine	Open ocean, Pacific	6943220	6359	6383
DK1_NH57388A	GCF_900069025	Clinical	Sputum	6212531	5610	5629
PAO1_Orsay	GCF_900070375	Laboratory		6276469	5682	5681
PA14Or_reads	GCF_900095805	Laboratory		6541482	5910	5897
PcyII-10	GCF_900149285	Clinical	Burn patient	6288645	5706	5687
C-NN2	GCF_900185255	Clinical	Cystic fibrosis lung	6902967	6601	6304

Table S2: Codon usage in the core genome of *P. aeruginosa*

Codon	Amino Acid	Fraction	Frequency	Number
GCA	A	0.038	4.396	704353
GCC	A	0.59	68.811	11024870
GCG	A	0.336	39.228	6285202
GCT	A	0.036	4.222	676495
TGC	C	0.91	9.129	1462659
TGT	C	0.09	0.905	144930
GAC	D	0.815	43.092	6904270
GAT	D	0.185	9.77	1565407
GAA	E	0.385	23.591	3779779
GAG	E	0.615	37.687	6038167
TTC	F	0.962	34.173	5475215
TTT	F	0.038	1.343	215176
GGA	G	0.045	3.725	596750
GGC	G	0.747	62.16	9959261
GGG	G	0.115	9.601	1538310
GGT	G	0.093	7.711	1235476
CAC	H	0.717	15.635	2505004
CAT	H	0.283	6.185	990964
ATA	I	0.018	0.726	116379
ATC	I	0.922	38.07	6099574
ATT	I	0.06	2.477	396868
AAA	K	0.117	3.311	530487
AAG	K	0.883	25.046	4012921
CTA	L	0.01	1.214	194550
CTC	L	0.225	28.361	4544058
CTG	L	0.675	84.919	13605836
CTT	L	0.022	2.795	447830
TTA	L	0.001	0.17	27304
TTG	L	0.067	8.416	1348387
ATG	M	1	20.435	3274077
AAC	N	0.871	22.215	3559341
AAT	N	0.129	3.288	526734
CCA	P	0.038	1.944	311541
CCC	P	0.26	13.269	2126027
CCG	P	0.666	33.985	5445057
CCT	P	0.036	1.857	297490
CAA	Q	0.143	6.098	976997
CAG	Q	0.857	36.403	5832556
AGA	R	0.004	0.314	50298
AGG	R	0.024	1.816	290882
CGA	R	0.028	2.142	343139
CGC	R	0.661	50.68	8119945
CGG	R	0.183	14.042	2249799
CGT	R	0.1	7.684	1231203
AGC	S	0.478	25.952	4158115
AGT	S	0.042	2.289	366725
TCA	S	0.008	0.414	66400
TCC	S	0.22	11.948	1914303
TCG	S	0.24	13.01	2084456
TCT	S	0.012	0.647	103604
ACA	T	0.015	0.598	95772
ACC	T	0.804	32.823	5258956
ACG	T	0.147	5.989	959603
ACT	T	0.035	1.413	226466
GTA	V	0.055	3.754	601448
GTC	V	0.422	29.019	4649462
GTG	V	0.49	33.675	5395491
GTT	V	0.033	2.283	365766
TGG	W	1	15.004	2403880
TAC	Y	0.803	20.121	3223868
TAT	Y	0.197	4.929	789765
TAA	*	0.091	0.281	45020
TAG	*	0.112	0.346	55481
TGA	*	0.797	2.462	394443

Table S3. Classification of inferred HGT genes to mobile genetic elements. To detect potential ICE, phage, IS or plasmid related genes, inferred HGT genes were queried against the ICEberg (Liu et al. 2019), PHASTER (Arndt et al. 2016) , ISfinder (Siguier et al. 2006) and PLSDB (Galata et al. 2018) databases, respectively. HGT genes with at least one hit with an e-value > 1e-5 were counted for each type of MGE. As a single HGT gene can sometimes be found on different MGE, the number of HGT genes on 'Any MGE' is slightly lower than the sum for separate MGE's.

Mobile Genetic Element	Number of HGT genes	% of HGT genes
Integrative conjugative elements (ICE)	427	6.7
Phages and prophages	1920	30
Insertion sequences (IS)	366	5.7
Plasmids	2118	33.1
Any MGE	4384	68.6

Table S4 HGT hotspots in the *P. aeruginosa* genome: List of *P. aeruginosa* core genes (indicated by their reference PAO1 locus tag) where adjacent HGT events were frequently detected. The last column indicates the number of HGT events that were found to be adjacent to each core gene. Only core genes with 20 or more adjacent HGT events were included in this table.

Core gene (PAO1 locus tag)	Annotation	Number of adjacent HGT events
PA2229	Hypothetical protein	141
PA0730	(R)-3-hydroxydecanoyl-ACP:CoA transacylase	122
PA2736.1	tRNA-Pro	121
PA2729	Hypothetical protein	120
PA0709	(4S)-4-hydroxy-5-phosphonooxypentane-2,3-dione isomerase	119
PA2216	Hypothetical protein	113
PA0826.2	tmRNA	101
PA0818	Hypothetical protein	100
PA4541	Heme/hemopexin-binding protein	89
PA4541.2	tRNA-Pro	89
PA0976	7-cyano-7-deazaguanine synthase	89
PA2820	Hypothetical protein	82
PA0988	1,4-dihydroxy-2-naphthoyl-CoA hydrolase	77
PA3824.1	tRNA-Leu	74
PA1961	HTH-type transcriptional regulator DmlR	74
PA1972	Phosphoethanolamine transferase EptA	74
PA3825	Putative cyclic-di-GMP phosphodiesterase AdrB	73
PA2817	Hypothetical protein	68
PA3619	Protein TolB	68
PA2583	Virulence sensor protein BvgS	67
PA2584	CDP-diacylglycerol—glycerol-3-phosphate-3-phosphatidyltransferase	67
PA3620	DNA mismatch repair protein MutS	67
PA1796.1	tRNA-Arg	66
PA1797a	Hypothetical protein	66
PA2793	Hypothetical protein	63
PA2795	tRNA-dihydrouridine synthase A	63
PA1013	Phosphoribosylaminoimidazole-succinocarboxamide synthase	63
PA1014	Poly-beta-1,6-N-acetyl-D-glucosamine synthase	63
PA3768	Multicopper oxidase mco	57
PA3769	GMP synthase	57
PA0256	Hypothetical protein	51
PA2570	PA-I galactophilic lectin	51
PA2571	Sensor protein ZraS	51
PA4674	putative HTH-type transcriptional regulator YbaQ	51
PA4673	Ribosome-binding ATPase YchF	48
PA2125	Glutarate-semialdehyde dehydrogenase DavD	47
PA2603	Thiosulfate sulfurtransferase GlpE	44
PA2603.1	tRNA-Ser	44
PA4138	Tyrosine--tRNA ligase	43
PA4139	Hypothetical protein	43
PA3663	Hypothetical protein	43
PA3664	Regulatory protein Spx	43
PA2581	Hypothetical protein	40
PA2581.1	tRNA-Cys	40
PA1934	Hypothetical protein	39
PA3141	UDP-N-acetyl-alpha-D-glucosamine C6 dehydratase	36
PA3161	Integration host factor subunit beta	33
PA3536	Hypothetical protein	30
PA3537	Ornithine carbamoyltransferase	30
PA4205	Hypothetical protein	29
PA4040	Hypothetical protein	27
PA4041	D(-)-tartrate dehydratase	24
PA1940	Hypothetical protein	23
PA4206	Efflux pump periplasmic linker BepF	23
PA2822	Free methionine-R-sulfoxide reductase	22
PA0961	Major cold shock protein CspA	21
PA1149	Hypothetical protein	21
